# Concurrent nutrient deficiencies are associated with dementia incidence

**DOI:** 10.1002/alz.13884

**Published:** 2024-06-12

**Authors:** Annick P. M. van Soest, Lisette C. P. G. M. de Groot, Renger F. Witkamp, Debora Melo van Lent, Sudha Seshadri, Ondine van de Rest

**Affiliations:** ^1^ Division of Human Nutrition and Health Wageningen University & Research Wageningen The Netherlands; ^2^ Glenn Biggs Institute for Alzheimer's & Neurodegenerative Diseases UT Health San Antonio San Antonio Texas USA; ^3^ Department of Neurology Boston University School of Medicine Boston Massachusetts USA; ^4^ The Framingham Heart Study Framingham Massachusetts USA

**Keywords:** 25‐hydroxyvitamin D, aging, Alzheimer's disease, apolipoprotein E, biomarkers, B vitamins, elderly, nutrition, older adults, polyunsaturated fatty acids, prevention

## Abstract

**INTRODUCTION:**

While observational research suggests a protective role for nutrition in brain aging, intervention studies remain inconclusive. This failing translation from observational to interventional research may result from overlooking nutrient interactions.

**METHODS:**

We developed a nutrient status index capturing the number of suboptimal statuses of omega‐3 fatty acids, homocysteine, and vitamin D (range 0 to 3). We associated this index with dementia incidence in a subsample (age ≥ 50 years) of the Framingham Heart Study Offspring cohort.

**RESULTS:**

Among 968 participants, 79 developed dementia over 15.5 years (median follow‐up). Each point increase in nutrient status index was associated with a 50% higher risk of dementia (hazard ratio [HR] = 1.50; 95% confidence interval [CI] = 1.16, 1.96). Participants with three high‐risk statuses had a four‐fold increased risk of dementia compared to participants without high‐risk status (HR = 4.68; 95% CI = 1.69, 12.94).

**DISCUSSION:**

Concurrent nutrient deficiencies are associated with the risk of dementia. The potential of optimizing nutritional status to lower dementia risk warrants further study.

**Highlights:**

- Nutrition and dementia research calls for multiple‐nutrient approaches.
- We studied combined suboptimal statuses of omega‐3 polyunsaturated fatty acids, homocysteine, and vitamin D.
- Suboptimal status of the three nutrients was associated with dementia risk.
- The risk estimate was larger than for other factors (ie, diabetes, apolipoprotein E ε4 carrier).
- Future studies should assess the effect of improving nutrient status on dementia risk.

## 1 INTRODUCTION

The rapidly increasing prevalence of dementia due to population aging, in combination with the enormous social impact and economic costs of dementia, demonstrate the urgent need for action. In the absence of curative treatment, the interest in preventive strategies is increasing.^1^

Preclinical research has indicated that several nutritional factors have the potential to modulate brain aging.^2^ Nutrients of interest include omega‐3 polyunsaturated fatty acids (n‐3 PUFAs), B vitamins, vitamin D, antioxidants, and polyphenols, which are mostly assumed to be effective due to their antioxidant, anti‐inflammatory, and vascular health‐promoting properties.^2^ Epidemiological research into the association between nutrient intake or status of single nutrients and brain aging generally confirms these preclinical findings.^2^ However, clinical trials involving single‐nutrient supplementation have yielded mainly negative results.^2^ This raises the question of which factors are responsible for the failure to translate findings from preclinical to clinical research.

A first explanation for the lack of effect of single‐nutrient supplementation in slowing brain aging is that nutrients are part of interacting processes with other nutrients quickly becoming limiting. Indeed, mechanisms underlying nutrition and brain aging are considered multifactorial,^3^ and evidence for dietary patterns is stronger than for single nutrients.^2^ A second explanation may be the lack of considering baseline nutrient status in the setup of clinical trials. In the majority of trials, participants are selected irrespective of their baseline nutrient status, while only individuals with a nutrient deficiency will likely benefit from nutrient supplementation.^4^ This is supported by a secondary analysis of the VITACOG trial, in which the effect of B‐vitamin supplementation on brain atrophy was dependent on baseline homocysteine levels.^5^

A better understanding of the cumulative beneficial effects of nutrients and baseline nutrient status may advance the field. However, the literature on multiple nutritional deficiencies in relation to brain aging is limited, with only two longitudinal studies having explored this topic. Here, it was demonstrated that a concurrent nutrient deficiency of n‐3 PUFAs, B vitamins, and vitamin D was associated with steep rates of cognitive decline,^6^ and combined suboptimal statuses of n‐3 PUFAs, carotenoids, and vitamin D were strongly associated with an increased risk of dementia.^7^ More longitudinal research is needed to reveal the complex interactions between multiple‐nutrient suboptimal statuses and cognitive aging. Specifically, the association between a combined suboptimal status of B vitamins, vitamin D, and n‐3 PUFAs with dementia incidence has not been investigated up till now. Therefore, we developed a nutrient status index including three nutrient biomarkers – homocysteine (as marker of vitamins B6 and B12 and folate status), vitamin D, and n‐3 PUFAs – and associated this index with dementia incidence in the Framingham Heart Study (FHS) Offspring cohort, a prospective community‐based cohort.

## 2 METHODS

### 2.1 Study design and population

The FHS is an ongoing prospective community‐based cohort of residents of the city of Framingham, Massachusetts, USA. In 1948, the Original cohort was established to gain insight into the factors contributing to cardiovascular disease.^8^ The Offspring cohort was established in 1971 as a second‐generation cohort, including children of the Original cohort and their spouses. A total of 5124 participants have been enrolled in the Offspring cohort. To date, these participants have been studied over 10 examination cycles, about once every 4 years.^9^ The study was approved by the Institutional Review Board of Boston University Medical Center, and all participants gave written informed consent.

For this study, we included data from participants aged ≥50 years, free of dementia, with available blood biomarker data on homocysteine, 25‐hydroxyvitamin D, and n‐3 PUFAs. We set the study baseline at exam 7, as biomarker data were measured at this time point. Among the 5124 participants in the FHS Offspring cohort at exam 7, 1525 participants had available data on all three biomarkers. All these participants were free of dementia at baseline. Data from 557 participants were excluded due to being younger than 50 years (*n* = 130) or missing covariate data (*n* = 427; of which *n* = 169 education; *n* = 25 apolipoprotein E (*APOE*) carrier status; *n* = 59 physical activity; *n* = 8 smoking; *n* = 107 alcohol intake; and *n* = 59 depression). Thus, our analysis included data of 968 participants.

### 2.2 Laboratory measurements

Fasting serum, plasma, and red blood cell samples were collected and stored at −80°C until testing. In the samples collected at exam 7, plasma total homocysteine concentration was measured by high‐performance liquid chromatography with fluorometric detection,^10^ and serum 25‐hydroxyvitamin D concentrations were determined by radioimmunoassay (DiaSorin, Stillwater, Minnesota, USA).^11^ In the samples collected at exam 8, the fatty acid composition of red blood cell membranes was determined by gas chromatography according to the methods described by Tan and colleagues.^12^ The omega‐3 index was calculated using the sum of eicosapentaenoic acid (EPA) and docosahexaenoic acid (DHA) and was expressed as a weight percentage of total fatty acids.

While it would be preferred to have fatty acid composition data also from exam 7, in line with the other biomarker data, we are confident that data from exam 8 are also valid. The fatty acids were measured in red blood cells, which is preferred over measurement in serum or plasma, as red blood cell fatty acid composition is more biologically stable^13^ and reflects dietary fatty acid intake over a longer time span (up to ∼120 days).^14^ Even though the time between exams 7 and 8 is longer than this time interval, we assume that the red blood cell measurement still provides a reliable and stable representation of the n‐3 PUFA status, as dietary patterns (and, thus, n‐3 PUFA intake) in the elderly are reasonably stable over time,^15^ and other factors that may influence variation (eg, geographic and genetic reasons) also have remained stable.

### 2.3 Ascertainment of incident dementia

Our outcome of interest was incidence of all‐cause dementia, assessed through December 2018. Extensive explanation of the diagnostic procedures used was published previously.^16^ In short, participants were continuously screened for cognitive decline. They were flagged for being at risk when they experienced a decline in routinely administered Mini–Mental State Examination performance, when participants, family members, or outside medical records reported subjective cognitive decline or when participants were referred for further screening by FHS staff or physicians. Subsequently, flagged participants underwent additional neuropsychological examination. A neurologist evaluated possible cognitive impairment or dementia and referred participants for dementia review. Dementia diagnosis was made by consensus of at least one neurologist and one neuropsychologist and was based on criteria from the *Diagnostic and Statistical Manual of Mental Disorders*, 4th edition.^17^ If a participant passed away or was lost to follow‐up, the review panel reviewed medical records up to the date of death/loss to follow‐up to determine whether the participant may have had cognitive decline.

**RESEARCH IN CONTEXT**

1. **Systematic review**: A literature review using PubMed regarding associations between concurrent nutrient deficiencies and brain aging yielded only two longitudinal studies. These studies show associations between combined suboptimal status of multiple nutrients with cognitive decline and dementia risk, with relatively large effect sizes. This necessitates further research.
2. **Interpretation**: We demonstrated that suboptimal status of one or more critical nutrients (omega‐3 polyunsaturated fatty acids, homocysteine, and vitamin D) was associated with dementia risk, with each additional suboptimal nutrient status elevating this risk. Individuals with suboptimal status for all three nutrients showed a four‐fold higher risk compared to those with optimal levels for all, confirming the potential of multiple‐nutrient strategies for the first time in a US population.
3. **Future directions**: Our findings can serve as the basis for designing future intervention trials in dementia prevention, where suboptimal nutrient statuses are selectively optimized, such as through personalized multinutrient supplementation or a brain‐healthy dietary intervention.

### 2.4 Covariates

Data for all covariates were collected at study baseline (exam 7). Information on age, sex, education level (no high school degree, high school degree, some college, or college degree), smoking status (never, former, or current) was obtained via medical questionnaires. *APOE* genotype was determined as described previously^18^ and classified into carriers and non‐carriers of at least one ε4 allele. Physical activity was self‐reported and measured by the physical activity index.^19^ Alcohol consumption was estimated from a food frequency questionnaire and classified as non‐excessive or excessive (< or ≥21 units per week). Hypertension was defined as systolic blood pressure >140 mmHg and/or use of antihypertensive medication, and diabetes was defined as random blood glucose ≥200 mg/dL or fasting blood glucose ≥126 mg/dL or on anti‐diabetic medication. Finally, depression was defined as a score of ≥16 on the Center for Epidemiologic Studies Depression Scale (CES‐D).^20^

### 2.5 Construction of nutrient status index

To construct the nutrient status index as used in our study, we combined the approaches of Bowman and colleagues^6^ and Neuffer and colleagues.^7^ This nutrient status index indicates the number of high‐risk statuses for three nutrients: homocysteine (as marker of B vitamin status), vitamin D, and n‐3 PUFAs. These nutrient biomarkers were selected a priori on the basis of having a plausible mechanism of action in preventing dementia, having proof from observational studies of the beneficial associations between the nutrient biomarker and dementia risk,^2^ and being readily available in the FHS Offspring cohort. The cut‐off for what level is high risk was based on our own data a posteriori. We did this by visualizing the dose‐response relationships between each nutrient and the risk of dementia, using penalized splines in a Cox proportional hazard model. Each model used age at exam 7 (delayed entry) and age at time of event or censoring (age as time scale), nutrient status winsorized at the 2.5th and 97.5th percentiles, and the covariates sex, education, and *APOE* ε4 carrier status. After visualization of the dose‐response relationships, we set cut‐offs based on graphical inspection of the curves where the splines crossed *y* = 0.

**FIGURE 1:**
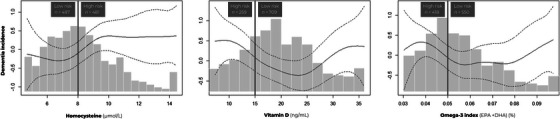
Dose‐response relationships between nutrient status of homocysteine, vitamin D, and omega‐3 polyunsaturated fatty acids and dementia risk, used to set cut‐offs for optimal (low risk) and suboptimal (high risk) status to construct the nutrient status index. EPA, eicosapentaenoic acid; DHA, docosahexaenoic acid.

Figure 1 shows the dose‐response associations between the individual nutrient statuses with dementia. For homocysteine, the cut‐off was set at 8 μmol/L. As homocysteine level was positively associated with dementia risk, participants with homocysteine status ≥8 μmol/L were classified in the high‐risk category, and those with status < 8 μmol/L were classified as low risk. The cut‐off for vitamin D (measured as 25‐hydroxyvitamin D) was set at 15 ng/mL (37.5 nmol/L). For vitamin D levels between 5 and 25 ng/mL (12.5‐62.5 nmol/L), there was an inverse association between vitamin D status and dementia incidence. To this end, participants with vitamin D levels ≤15 ng/mL were classified as high risk and participants with a status >15 ng/mL as low risk. Even though the line *y* = 0 also crosses the spline at vitamin D level 28 ng/mL, we did not set another cut‐off because very few participants had vitamin D levels of ≥28 ng/mL, and this observation cannot be explained from a physiological perspective. For omega‐3 PUFAs, the cut‐off was set at an omega‐3 index of 5%. As the omega‐3 index was inversely associated with dementia incidence, participants with an omega‐3 index ≤5% were classified as high risk and participants with status > 5% as low risk. Again, while *y* = 0 also crosses the spline at omega‐3 index 8.5%, we did not set a second cut‐off for reasons given earlier.

The nutrient status index captures the number of high‐risk statuses (range 0 to 3). In other words, we assigned a score of 0 if a participant fell into the low‐risk category and a score of 1 if the participant was at high risk. The ultimate nutrient status index sums the values for homocysteine, vitamin D, and n‐3 PUFA status.

### 2.6 Statistical analysis

For the comparison of baseline characteristics, participants were grouped according to the number of high‐risk nutrient statuses. Baseline characteristics for these groups were compared using ANOVA or Kruskal–Wallis test for continuous variables and chi‐squared for categorical variables.

For the main analyses, we examined the longitudinal association between the nutrient status index and dementia incidence. We performed multivariate‐adjusted Cox proportional hazard models and modeled delayed entry and age as time scale as a function of the nutrient status index (categorical and continuous). Model 1 was adjusted for the covariates age, sex, education, and *APOE* ε4 carrier status, and Model 2 was additionally adjusted for physical activity, smoking, alcohol intake, hypertension, diabetes, and depression. The proportional hazard assumption was met. Results are presented as adjusted hazard ratios (HRs) accompanied by 95% confidence intervals (CIs). The HRs represent the difference in dementia risk compared to participants without a high‐risk status (categorical) and the change in dementia risk by each unit increase in the nutrient status index (continuous).

For a sensitivity analysis, we evaluated the robustness of the nutrient status index. We tested the effect of adjusting the cut‐offs by 10% by adopting the same cut‐offs as in a previous article,^6^ changing the definition of omega‐3 PUFA status by including docosapentaenoic acid (DPA) and by changing the age cut‐off at ≥60 years. The nutrient status indices thus obtained were again associated with dementia incidence similarly to the primary analysis. Additionally, to investigate whether sex or *APOE* ε4 carrier status modified the association, we tested for interactions with these two variables.

A *p*‐value < 0.05 and < 0.10 were considered statistically significant for the main analyses and for the tests for interactions, respectively. All analyses were performed using RStudio version 1.1.463.^21^

## 3 RESULTS

### 3.1 Participant characteristics

Baseline characteristics of the 968 participants are presented in Table 1 and the prevalence of high‐risk status in Table S1. The participants were on average 61.4 ± 7.6 years, and 48% were male. A total of 22% of participants were carriers of at least one *APOE* ε4 allele. The nutrient status index ranged from 0 (low risk for all nutrients) to 3 (high risk for all nutrients), with the majority of participants (40%) having one high‐risk nutrient status. Participants with a higher number of high‐risk statuses were more likely to be male, had on average a higher body mass index, and were more often current or former smoker.

**TABLE 1:** Characteristics of the Framingham Heart Study population per number of high‐risk statuses.

		Number of high‐risk statuses				
	Overall (*n* = 968)	0 (*n* = 232)	1 (*n* = 391)	2 (*n* = 268)	3 (*n* = 77)	*p*‐value
Dementia cases *n* (%)	79 (8%)	7 (3%)	38 (10%)	25 (9%)	9 (12%)	
Age (years)	61.4 ± 7.6	60.8 ± 7.1	61.4 ± 7.8	62.1 ± 7.7	61.1 ± 7.8	.33
Sex, *n* (%)						<.001
Male	461 (48%)	75 (32%)	185 (47%)	159 (59%)	42 (55%)	
Female	507 (52%)	157 (68%)	206 (53%)	109 (41%)	35 (54%)	
*APOE* ε4 carrier *n* (%)	215 (22%)	50 (22%)	85 (22%)	60 (22%)	20 (26%)	.85
Level of education, *n* (%)						<.001
High school non‐graduate	34 (4%)	4 (2%)	9 (2%)	18 (7%)	3 (4%)	
High school graduate	313 (32%)	66 (28%)	126 (32%)	94 (35%)	27 (35%)	
Some college	238 (25%)	70 (30%)	81 (21%)	67 (25%)	20 (26%)	
College graduate	383 (40%)	92 (40%)	175 (45%)	89 (33%)	27 (35%)	
BMI (kg/m^2^)	28.1 ± 5.1	26.4 ± 4.3	28.3 ± 5.1	28.9 ± 5.1	29.8 ± 5.4	<.001
Physical activity (PAI score)	38.2 ± 6.4	38.7 ± 5.9	37.7 ± 6.3	38.6 ± 7.1	37.9 ± 5.9	.18
Smoking behavior, *n* (%)						.02
Current smoker	30 (3%)	3 (1%)	9 (2%)	13 (5%)	5 (6%)	
Former smoker	563 (58%)	131 (56%)	229 (59%)	151 (56%)	53 (69%)	
Never‐smoker	374 (39%)	98 (42%)	153 (39%)	104 (39%)	19 (25%)	
Systolic blood pressure (mmHg)	126 ± 17	124 ± 17	127 ± 17	127 ± 17	127 ± 16	.07
Use of anti‐hypertensives *n* (%)	337 (35%)	70 (30%)	148 (38%)	94 (35%)	25 (32%)	.26
Depression (CES‐D)	3 [0 – 6]	2 [1 – 7]	3 [0 – 6]	2 [0 – 6]	3 [1 – 7]	.42
Cardiovascular disease, *n* (%)	114 (12%)	20 (9%)	53 (14%)	33 (12%)	8 (10%)	.30
Diabetes, *n* (%)	84 (9%)	13 (6%)	31 (8%)	32 (12%)	8 (10%)	.07
Omega‐3 index (wt%)	5.6 ± 1.7	6.8 ± 1.5	5.7 ± 1.7	4.7 ± 1.2	4.0 ± 0.7	<.001
Plasma homocysteine (μmol/L)	8.4 ± 3.8	6.4 ± 1.1	8.1 ± 2.3	9.5 ± 2.5	11.7 ± 9.9	<.001
Serum 25‐hydroxyvitamin D (ng/mL)	19.9 ± 7.7	23.7 ± 6.7	20.7 ± 6.8	18.1 ± 7.9	10.7 ± 2.8	<.001

*Note*: Data are mean ± standard deviation, median [IQR] or number (%).

Abbreviations: *APOE*, Apolipoprotein E; BMI: body mass index; CES‐D, Center for Epidemiologic Studies Depression Scale; PAI, Physical Activity Index.

### 3.2 Association nutrient status index with dementia incidence

Among the 968 participants, 79 developed dementia over a median follow‐up of 15.5 [12.9, 19.0] years. In multivariable‐adjusted models, the nutrient status index was associated with dementia incidence (Table 2). Each point increase in nutrient status index was associated with a 50% higher risk of dementia (HR = 1.50; 95% CI = 1.16, 1.96). Moreover, participants with three high‐risk statuses had a four‐fold increased risk of dementia compared to participants without a high‐risk status (HR = 4.64; 95% CI = 1.68, 12.83).

**TABLE 2:** Risk of dementia by multinutrient status index.

	Crude model	Model 1	Model 2
Number of high‐risk statuses			
0 (lowest risk)	Reference	Reference	Reference
1	2.98 [1.33, 6.70] 0.008	2.79 [1.23, 6.33] 0.014	2.89 [1.27, 6.58] 0.012
2	3.03 [1.31, 7.00] 0.010	3.09 [1.32, 7.24] 0.009	3.28 [1.38, 7.80] 0.007
3 (highest risk)	4.30 [1.60, 11.56] 0.004	4.70 [1.74, 12.69] 0.002	4.68 [1.69, 12.94] 0.003
Continuous	1.43 [1.11, 1.83] 0.005	1.48 [1.15, 1.93] 0.002	1.50 [1.16, 1.96] 0.002

*Note*: Data are hazard ratio [95% confidence interval] *p*‐value. Model 1: adjusted for age, sex, education, and *APOE* ε4 carrier status; Model 2: additionally adjusted for physical activity, smoking, alcohol consumption, diabetes, hypertension, and depression. In the continuous analysis, data are shown per point increment in nutrient status index.

### 3.3 Sensitivity analyses

We performed sensitivity analyses to assess the robustness of the association between the nutrient status index with dementia incidence. Overall, the association was robust to changes in nutrient status cut‐offs (Table S2). Varying the cut‐offs by 10% and adopting the same cut‐offs as Bowman and colleagues^6^ did not alter the results. Similarly, results were robust to variations in study population and components. Changing the age cut‐off to ≥60 years or adapting the definition of the omega‐3 index by including DPA in addition to EPA and DHA did not change the results.

Subsequently, we tested for interactions to investigate whether sex and *APOE* ε4 carrier status modified the association between the nutrient status index and dementia incidence (Table S3). Interestingly, *APOE* ε4 carrier status appeared to influence the association (*p*_interaction _= 0.01). The nutrient status index (continuous) was positively associated with dementia incidence in carriers (HR_per point increase _= 2.05, 95% CI = 1.23, 2.44), but not in non‐carriers (HR_per point increase _= 1.11, 95% CI = 0.77, 1.59). There was no significant overall interaction between sex and nutrient status index (*p* = .23).

To put our found effect size for the nutrient status index into context, we assessed how many known risk factors increased the risk of developing dementia in our sample. The risk of dementia was doubled for current smokers (HR = 1.97; 95% CI = 0.53, 7.32) and for individuals with diabetes (HR = 2.18; 95% CI = 1.14, 4.17) and tripled for carriers of the *APOE* ε4 allele (HR = 3.11; 95% CI = 1.92, 5.05) (Table S4).

## 4 DISCUSSION

Using the nutrient status index developed for our study, we found that individuals with a higher index, that is, having suboptimal status of homocysteine (as a marker of B vitamins), vitamin D, and n‐3 PUFAs, had a higher risk of developing dementia compared to those with a lower index. Remarkably, a suboptimal status of all three nutrients was associated with a four‐fold increased risk of dementia compared to individuals without suboptimal statuses. In addition, *APOE* ε4 carrier status appeared to influence the association between nutrient status index and dementia incidence, with the association only evident in *APOE* ε4 carriers.

The effect size we observed was substantial: a four‐fold increased risk of developing dementia in individuals with combined suboptimal status of n‐3 PUFAs, vitamin D, and homocysteine. This effect size is large in comparison with other risk factors of dementia. In our sample, being a current smoker or having diabetes doubled the risk, and being a carrier of at least one *APOE* ε4 allele tripled the risk of dementia.

Previous research complements our results and the large effect sizes. To our knowledge, the association between multiple‐nutrient suboptimal status and brain aging has been investigated in two other studies. In a secondary analysis of the Bordeaux Three‐City (3C) study, Neuffer and colleagues developed a nutrient status index composed of n‐3 PUFAs (EPA+DHA+DPA), carotenoids, and 25(OH)D. Similarly to our approach, they set nutrient cut‐offs based on their own data (n3‐PUFA at 3 and 4.5%, carotenoids at 100 and 200 μg/mmol, and vitamin D at 8 and 26 ng/mL). A higher nutrient status index (ie, more nutrient suboptimal statuses) was associated with a higher risk to develop dementia. The 13% of participants with the highest nutrient status index had a four‐fold increased chance of developing dementia compared to the 21% with the lowest index scores.^7^ Additionally, Bowman and colleagues investigated the role of combined deficiencies in n‐3 PUFAs (EPA + DHA), 25(OH)D, and homocysteine on cognitive decline in a secondary analysis of the French Multi‐domain Alzheimer's Prevention Trial (MAPT), with cut‐offs for nutrient deficiencies set a priori (n‐3 PUFA 4.82%, Hcy 14 μmol/L, vitamin D 20 ng/mL). Individuals without nutrient deficiencies of these nutrients showed cognitive improvements over 3 years, while each additional nutrient deficiency led to an incremental faster rate of cognitive decline.^6^

While this earlier research also demonstrated associations between multiple‐nutrient suboptimal statuses and brain aging, direct comparison between results is complicated by differences in components and cut‐offs.

Regarding components, in the 3C study data were available on carotenoid but not on homocysteine status. Carotenoids are also nutrients of prime interest in relation to the aging brain. These nutrients reduce oxidative stress, a mechanism involved in the pathogenesis of dementia.^22^ Additionally, higher carotenoid status has been associated with lower odds of dementia.^23^ It is a limitation of the current study that we did not have data available on carotenoid status or on other antioxidant nutrient statuses like vitamin C or E. Further research on multinutrient suboptimal statuses should consider incorporating antioxidant nutrients, alongside n‐3 PUFAs, homocysteine, and vitamin D status.

With respect to cut‐offs, our homocysteine cut‐off (8 μmol/L) was lower compared to the French MAPT trial (14 μmol/L), which was anticipated because of folate fortification in the United States. Our low cut‐off is likely not applicable in countries where foods are not fortified. Vitamin D cut‐offs among studies varied, ranging from 15 ng/mL in FHS, 20 ng/mL in MAPT, to 8 and 26 ng/mL in 3C. Our cut‐off is lower than the World Health Organization guidelines of 20 ng/mL (50 nmol/L). However, this cut‐off was set for bone health rather than brain health. Our n‐3 PUFA cut‐off (5%) was slightly higher compared to the 3C study (3 and 4.5%) and MAPT trial (4.82%) but still low compared to the target range of 8% to 11%.^24^ While increasing the cut‐off to this target range could provide even stronger protective associations, the baseline omega‐3 index of our study population was too low to ensure sufficient contrast. All in all, optimal nutrient cut‐offs may be population‐specific, and therefore, it can be seen as a limitation that we set cut‐offs based on our own data. Nevertheless, the nutrient status index applied in our study was robust to variations in cut‐offs, as demonstrated in the sensitivity analyses, and this adds to the robustness of our findings. In addition, despite methodological differences between our and previous studies, results are remarkably consistent.

The observation that there is an association between multiple suboptimal nutrient statuses and dementia risk is biologically plausible. Preclinical studies stress that brain aging depends on multiple, dynamically interacting mechanisms, with nutrients playing distinctive roles. Vitamin D, among others, promotes healthy brain aging by suppressing amyloid beta deposition, regulating calcium homeostasis, and reducing oxidative stress and inflammation.^25^ Omega‐3 fatty acids also possess anti‐inflammatory and antioxidant properties, as well as vascular health‐promoting effects. In addition, these fatty acids serve as building blocks for neuronal tissue.^26^ Homocysteine has been shown to negatively impact the aging brain by impairing vascular functioning and increasing tau phosphorylation and via inhibition of methylation reactions.^27^ Considering the multifactorial nature of dementia, it is conceivable that these single effects of the nutrients targeting different mechanisms of action have additive effects.

In addition to these additive effects, it is possible that nutrients act in a synergistic manner. This has been hypothesized for homocysteine and n‐3 PUFAs, as a consequence of the regulatory role of homocysteine in the transport of n‐3 PUFAs to the brain.^28^ DHA is transported with the help of phosphatidylcholine (PC), the formation of which is dependent on homocysteine levels. Elevated homocysteine levels decrease the activity of phosphatidylethanolamine N‐methyltransferase, the enzyme responsible for the conversion of phosphatidylethanolamine to PC. Consequently, this results in low transport of n‐3 PUFAs to the brain.^28^ Indeed, the synergistic effects between homocysteine and n‐3 PUFAs have been confirmed in secondary analyses of B vitamin^29^, ^30^, ^31^ and n‐3 PUFA^32^ supplementation trials.

For further research, we strongly encourage researchers with access to data on multiple‐nutrient statuses and brain aging outcomes to further investigate the potential of multinutrient suboptimal statuses. This will give more insight into optimal nutrient status cut‐offs. Additionally, these results can be the basis for the design of clinical trials, in which the nutrient status index can be used to select participants at nutritional risk of dementia.^4^ Participants may then undergo nutrient supplementation to correct suboptimal status. Instead of nutrient supplementation, participants could undergo a diet intervention targeted at improving general dietary intake, as suboptimal status of the three nutrients investigated in this research could also be a proxy for general suboptimal nutritional status.

Another topic that deserves further investigation is the interaction with the *APOE* ε4 genotype. We observed an interaction with this genotype, with strong associations between multinutrient suboptimal statuses and dementia in carriers, but not in non‐carriers of the *APOE* ε4 allele. This interaction has not been investigated in the two previous articles on multinutrient suboptimal statuses.^6^, ^7^ However, a large body of literature is available on the interaction between *APOE* genotype and n‐3 PUFAs in relation to brain aging. According to this literature, *APOE* ε4 carriers seem more susceptible to the benefits of omega‐3 PUFAs in preclinical stages, while benefits in the clinical stages are limited to non‐carriers.^33^ The mechanistic rationale on why *APOE* ε4 carriers may need more n‐3 PUFAs during the preclinical stage is that they experience accelerated DHA catabolism and less efficient transport of DHA both across the blood brain barrier and within the brain. These processes occur before the onset of neurodegeneration.^33^ At baseline, our study population was likely in the preclinical stage of dementia, considering the relatively young age (≥50 years). Literature on vitamin D and homocysteine in relation to the *APOE* genotype is limited, but a pattern similar to that of n‐3 PUFAs has been demonstrated for other preventive strategies to lower dementia risk, with *APOE* ε4 carriers benefitting more in preclinical stages.^34^ However, it is important to emphasize that these results come from a subgroup analysis, so interpretation is limited. Further research is required to confirm the interaction with the *APOE* ε4 genotype.

In conclusion, in our community‐based sample, concurrent suboptimal status of n‐3 PUFAs, homocysteine, and vitamin D is associated with the risk of dementia. The results support earlier observations that multiple‐nutrient suboptimal status is highly detrimental for brain aging, suggesting that nutrition is a key modifiable risk factor for dementia. Further research is needed to optimize nutrient status cut‐offs and to study the potential of optimizing nutritional status to lower dementia risk.

## CONFLICT OF INTEREST STATEMENT

Dr. Melo van Lent is chair of the Alzheimer's Association ISTAART Nutrition Metabolism and Dementia Professional Interest Area. All other authors report no conflicts of interest. Author disclosures are available in the supporting information.

## CONSENT STATEMENT

All participants provided written informed consent. The study protocol was approved by the Institutional Review Board at Boston University Medical Center.

## Supporting information

Supporting Information

Supporting Information
