# Supplementary material for: Concurrent nutrient deficiencies are associated with dementia incidence
Source: Alzheimers Dement. 2024 Jun 12;20(7):4594–601. doi: 10.1002/alz.13884 (PMC11247665; doi:10.1002/alz.13884)
Supplement: Supplementary file 1 — Supporting Information [file ALZ-20-4594-s001.docx]

**Supplementary materials**

**Concurrent nutrient deficiencies are associated with dementia incidence**

Annick P.M. van Soest^1^, Lisette C.P.G.M. de Groot^1^, Renger F. Witkamp^1^, Debora Melo van Lent^2,3,4^, Sudha Seshadri^2,3,4^, Ondine van de Rest^1^

^1^ Division of Human Nutrition and Health, Wageningen University & Research, Wageningen, The Netherlands

^2^ Glenn Biggs Institute for Alzheimer's & Neurodegenerative Diseases, UT Health San Antonio, San Antonio, TX, United States

^3^ Department of Neurology, Boston University School of Medicine, Boston, MA, United States

^4^ The Framingham Heart Study, Framingham, MA, United States

Corresponding author: Annick van Soest, P.O. Box 12 6700 AA Wageningen The Netherlands, +31 317 488 077, annick.vansoest@wur.nl

| Nutrient(s) with high risk status | | Sample size, n (%) | |
| --- | --- | --- | --- |
| 0 risk statuses (lowest risk) | | 232 (24%) | |
| 1 risk status | | 391 (40%) | |
|  | Hcy |  | 186 (19%) |
|  | Vit D |  | 67 (7%) |
|  | n-3 PUFA |  | 138 (14%) |
| 2 risk statuses | | 268 (28%) | |
|  | Hcy & vit D |  | 65 (7%) |
|  | Hcy & n-3 PUFA |  | 153 (16%) |
|  | Vit D & n-3 PUFA |  | 50 (5%) |
| 3 risk statuses (highest risk) | | 77 (8%) | |

**Supplementary table 1:** Overview of the prevalence of high risk nutrient statuses of homocysteine, vitamin D and omega-3 polyunsaturated fatty acids

|  | Effect size | | | |
| --- | --- | --- | --- | --- |
|  | Crude | Model 1 | Model 2 | p-value |
| Adapting data-based cut-offs | | | | |
| Homocysteine | | | | |
| Cut-off 10% lower | 1.40 [1.07, 1.84] | 1.45 [1.10, 1.92] | 1.44 [1.08, 1.93] | 0.01 |
| Cut-off 10% higher | 1.40 [1.10, 1.79] | 1.42 [1.10, 1.83 | 1.43 [1.10, 1.85] | 0.008 |
| Vitamin D | | | | |
| Cut-off 10% lower | 1.53 [1.18, 1.97] | 1.61 [1.23, 2.10] | 1.64 [1.25, 2.16] | <0.001 |
| Cut-off 10% higher | 1.37 [1.08, 1.74] | 1.43 [1.11, 1.83] | 1.45 [1.12, 1.87] | 0.004 |
| Omega-3 index | | | | |
| Cut-off 10% lower | 1.46 [1.13, 1.88] | 1.53 [1.18, 1.98] | 1.55 [1.19, 2.02] | 0.001 |
| Cut-off 10% higher | 1.41 [1.10, 1.81] | 1.46 [1.14, 1.88] | 1.44 [1.11, 1.86] | 0.006 |
| Adapting literature-based cut-offs | | | | |
| Cut-offs according to Bowman 2019^1^ | 1.49 [1.10, 2.01] | 1.45 [1.06, 1.98] | 1.41 [1.03, 1.94] | 0.03 |
| Changing definition omega-3 index | | | | |
| Including DPA | 1.42 [1.10, 1.83] | 1.49 [1.15, 1.94] | 1.48 [1.13, 1.93] | 0.005 |
| Changing age cut-off | | | |  |
| ≥60y | 1.42 [1.09, 1.83] | 1.48 [1.13, 1.93] | 1.48 [1.13, 1.94] | 0.004 |

Abbreviations: Hcy: homocysteine; n-3 PUFA: omega-3 polyunsaturated fatty acid; vit D: vitamin D

**Supplementary table 2**: Association between nutrient status index and dementia incidence following changes in definitions exposures and cut-offs

Data are HR [95% CI] per point increase in nutrient status index. Model 1: adjusted for age, sex, education, and ApoE4 carrier status; Model 2: additionally adjusted for physical activity, smoking, alcohol consumption, diabetes, hypertension, and depression.
^1^ [6]

|  | Effect size | | | | Overall interaction |
| --- | --- | --- | --- | --- | --- |
|  | Crude | Model 1 | Model 2 | p-value | p-value |
| ApoE4 carrier status | | | | | |
| Carrier  (n=215, of which 33 dementia cases) | 2.10 [1.42, 3.10] | 2.14 [1.42, 3.22] | 2.05  [1.23, 2.44] | 0.001 | 0.01 |
| Non-carrier  (n=753, of which 46 dementia cases) | 1.13 [0.81, 1.57] | 1.10 [0.79, 1.55] | 1.11  [0.77, 1.59] | 0.57 |  |
| Sex | | | | | |
| Female (n=507, of which 40 dementia cases) | 1.55  [1.11, 2.15] | 1.62 [1.17, 2.25] | 1.74  [1.23, 2.44] | 0.002 | 0.23 |
| Male  (n=461, of which 39 dementia cases) | 1.23 [0.83, 1.82] | 1.28 [0.85, 1.93] | 1.27  [0.82, 1.94] | 0.28 |  |

**Supplementary table 3**: Association between nutrient status index and dementia incidence, stratified by sex and ApoE4 carrier status

Data are HR [95% CI] per point increase in nutrient status index. Model 1: adjusted for age, education, and ApoE4 carrier status or sex; Model 2: additionally adjusted for physical activity, smoking, alcohol consumption, diabetes, hypertension, and depression.

|  | Effect size | | | |
| --- | --- | --- | --- | --- |
|  | Crude model | Model 1 | Model 2 | p-value |
| Current smoker | 2.03 [0.62, 6.65] | 2.79  [0.80, 9.64] | 1.97  [0.53, 7.32] | 0.31 |
| Diabetes | 2.03  [1.13, 3.64] | 2.04  [1.13, 3.71] | 2.18  [1.14, 4.17] | 0.02 |
| ApoE4 carrier | 2.91  [1.86, 4.56] | 2.89  [1.84, 4.56] | 3.11  [1.92, 5.05] | <0.001 |

**Supplementary table 4**: Association between smoking, diabetes and ApoE4 carrier status and dementia incidence

Data are HR [95% CI]. Model 1: adjusted for age, education, and ApoE4 carrier status. Model 2: additionally adjusted for nutrient status index, physical activity, smoking, alcohol consumption, diabetes, hypertension, and depression.
